# Comparative effectiveness trial of transoral head and neck surgery followed by adjuvant radio(chemo)therapy versus primary radiochemotherapy for oropharyngeal cancer (TopROC)

**DOI:** 10.1186/s12885-020-07127-2

**Published:** 2020-07-29

**Authors:** Lara Bußmann, Simon Laban, Claus Wittekindt, Carmen Stromberger, Silke Tribius, Nikolaus Möckelmann, Arne Böttcher, Christian Stephan Betz, Jens Peter Klussmann, Volker Budach, Adrian Muenscher, Chia-Jung Busch

**Affiliations:** 1 Department of Otorhinolaryngology and Head and Neck Surgery, University Medical Center Hamburg Eppendorf, Martinistrasse 52, 20246 Hamburg, Germany; 2 Department of Otorhinolaryngology and Head and Neck Surgery, University Medical Center Ulm, Ulm, Germany; 3 Department of Otorhinolaryngology and Head and Neck Surgery, University Medical Center Gießen, Gießen, Germany; 4 Department of Radiation Oncology, Charité University Medicine Berlin, Berlin, Germany; 5 Hermann-Holthusen-Institut for Radiation Oncology, Asklepios Klinik St. Georg, Hamburg, Germany; 6 Department of Otorhinolaryngology, Head and Neck Surgery, Faculty of Medicine and University Hospital Cologne, University of Cologne, Cologne, Germany

**Keywords:** Head and neck cancer, Oropharynx, Radiotherapy, Transoral surgery, Survival, Quality of life, Comparative effectiveness trial, Randomized controlled trial

## Abstract

**Background:**

For loco-regionally advanced, but transorally resectable oropharyngeal cancer (OPSCC), the current standard of care includes surgical resection and risk-adapted adjuvant (chemo) radiotherapy, or definite chemoradiation with or without salvage surgery. While transoral surgery for OPSCC has increased over the last decade for example in the United States due to transoral robotic surgery, this treatment approach has a long history in Germany. In contrast to Anglo-Saxon countries, transoral surgical approaches have been used frequently in Germany to treat patients with oro-, hypopharyngeal and laryngeal cancer. Transoral laser microsurgery (TLM) has had a long tradition since its introduction in the early 70s. To date, the different therapeutic approaches to transorally resectable OPSCC have not been directly compared to each other in a randomized trial concerning disease control and survival. The goal of this study is to compare initial transoral surgery to definitive chemoradiation for resectable OPSCC, especially with regards to local and regional control.

**Methods:**

TopROC is a prospective, two-arm, open label, multicenter, randomized, and controlled comparative effectiveness study. Eligible patients are ≥18 years old with treatment-naïve, histologically proven OPSCC (T1, N2a-c, M0; T2, N1–2c, M0; T3, N0-2c, M0 UICC vers. 7) which are amenable to transoral resection. Two hundred eighty patients will be randomly assigned (1:1) to surgical treatment (arm A) or chemoradiation (arm B). Standard of care treatment will be performed according to daily routine practice. Arm A consists of transoral surgical resection with neck dissection followed by risk-adapted adjuvant therapy. Patients treated in arm B receive standard chemoradiation, residual tumor may be subject to salvage surgery. Follow-up visits for 3 years are planned. Primary endpoint is time to local or locoregional failure (LRF). Secondary endpoints include overall and disease free survival, toxicity, and patient reported outcomes. Approximately 20 centers will be involved in Germany. This trial is supported by the German Cancer Aid and accompanied by a scientific support program.

**Discussion:**

This study will shed light on an urgently-needed randomized comparison of the strategy of primary chemoradiation vs. primary surgical approach. As a comparative effectiveness trial, it is designed to provide data based on two established regimens in daily clinical routine.

**Trial registration:**

NCT03691441 Registered 1 October 2018 - Retrospectively registered.

## Background

In contrast to other head and neck cancers, the incidence of oropharyngeal cancer (OPSCC) has increased significantly in many countries in the last decades, including the USA and Europe. This increase is largely attributed to the rise in the human papillomavirus (HPV)-related subgroup of oropharyngeal cancer (HPV + -OPSCC) [1–4]. The range of HPV-positive OPSCC shows considerable variation depending on tumor site and geographical origin of the patients in Germany [5]. An analysis of the German Cancer Consortium Radiation Oncology Group (DKTK-ROG) demonstrated 48% HPV-DNA positivity and 53,3% p16 positivity in their cohort derived from 8 centers [6]. A single center analysis showed 28% positivity for HPV-DNA and p16 [7], whereas another analysis of 8 health care centers, mostly from Northern Germany, showed an overall HPV-DNA prevalence rate of 23.5% [8]. Besides HPV-status, nicotine and alcohol abuse are still major prognostic factors. A comparison demonstrated 2-year survival rates of 98% versus 74% for never/ex-smokers compared to current smokers with HPV-positive OPSCC [6]. HPV-positive-OPSCC in patients with a history of years or decades of smoking presumably have substantially different biology compared to those from light or non-smokers [9]. However the prognostic significance of smoking is less clear in primary surgical treated patients [10].

The standard of care for OPSCC currently depends on the stage of the disease, as well as on patients’ and clinicians’ preferences. The multidisciplinary treatment portfolio of advanced head and neck cancer is based on three modalities: surgery, radiotherapy and systemic therapy. According to most guidelines, these modalities may be combined in a multimodality concept for the treatment of loco-regionally advanced head and neck tumors in the first place. For these diseases, the standard of care includes surgical resection with or without reconstruction and adjuvant risk-adapted chemoradiation, or definitive chemoradiation potentially followed by salvage surgery. The treatment guidelines of the National Comprehensive Cancer Network (NCCN) in the U.S., as well as those of the European Society for Medical Oncology (ESMO) in Europe also share this joint concept of equally having a surgical as well as a radiation-based approach [11, 12]. However, in some patients with resectable tumors, the poor anticipated functional outcome and/or the prognosis may not justify mutilating surgery and definite chemoradiation may be preferred. In terms of treatment recommendations, current therapeutic guidelines do not differentiate between HPV-positive and HPV-negative populations of patients with OPSCC yet. At this stage, international de-escalation trials are ongoing to determine whether HPV-positive-OPSCC may be subject to such recommendations [13]. Until now, in the setting of definitive radiotherapy, de-escalation trials show inferior survival rates. Thus radiotherapy with cisplatin still remains the standard of care in in HPV-positive as well as HPV-negative OPSCC [14, 15].

In contrast to the Anglo-Saxon countries, transoral surgical approaches have been used frequently in Germany to treat patients with oro-, hypopharyngeal and laryngeal primary [16]. Transoral laser microsurgery (TOLM or TLM) has had a long tradition since its first applications in the early 1970s. However, only a few multicenter studies and no prospective controlled trials have been performed to date [17, 18]. Since the introduction of robotic surgery in head and neck in the mid-2000s and its FDA-approval in 2009 (transoral robotic surgery: TORS), transoral surgical approaches are being increasingly used worldwide. In contrast to endoscopic laser surgery with a 2-dimensional view this new technology makes en bloc tumor resection more feasible with the advantage of an optimal 3-dimensional wide angle view [19, 20]. To date, only data from one prospective comparative trial have been presented, that shows better functional outcomes after radiotherapy than surgery [21]. The question is also, which of these two standard therapies is more effective in the daily clinical practice, including all specific conditions of non-ideal patients and our health care system. For this reason, a study concept other than a traditional randomized controlled trial is necessary to evaluate these state of the art therapies. Comparative effectiveness trials are pragmatic trials that focus on effectiveness (i.e., the benefit the treatment produces in routine clinical practice) and not on the efficacy (i.e., the benefit the treatment produces in an artificial environment) [22, 23].

The goal of this study is to compare primary surgery to primary radiation therapy for locally advanced, but transorally resectable oropharyngeal cancer with regards to local and locoregional control, survival, toxicity, quality of life and cost-effectiveness. Both treatment options represent state of the art procedures worldwide. To date, lack of randomization has been the most important limitation of most published data concerning treatment strategies in oropharyngeal cancer. To generate level Ib evidence, randomization between the two standard treatments is of indispensable importance. Results of this study may potentially be practice changing.

## Methods/design

This clinical trial has been approved by the ethics committee of the Ärztekammer in Hamburg (PVN5578). It is conducted in agreement with the ICH Harmonized Tripartite Guideline on Good Clinical Practice, valid since 17.01.1997, the Declaration of Helsinki (in its current version) and the respective national laws (in its current version).

It is a prospective, two-arm, open label, multicenter, randomized, controlled comparative effectiveness study.

The trial is based on an event-driven design: the final analysis will be performed when all events have been observed or the study was terminated at one of the interim analyses.

### Objectives

**Fig. 1:**
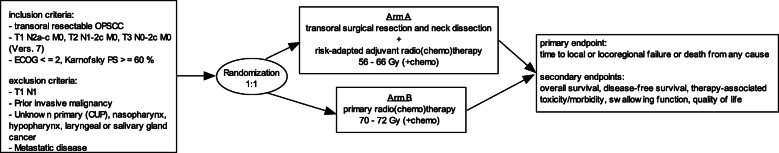
Study overview

The primary objective of this study is to evaluate the effectiveness of primary surgical versus non-surgical treatment in patients with locally advanced, but transorally resectable oropharyngeal cancer in terms of time to local or locoregional failure or death from any cause (see Fig. 1).

#### Primary endpoint

Time to local or locoregional failure or death from any cause (LRF):

Defined as time from randomization to date of first observed treatment failure confirmed by histologically proven tumor persistence or recurrence (either locally or locoregionally) or death from any cause whatever occurs first. In arm B, post-treatment residual primary tumor or neck nodes may be subject to salvage surgery within 10–14 weeks after completion of radiation treatment. Positive primary and/or neck specimens will be considered as local and/or locoregional failures.

#### Secondary endpoints

- Overall survival (OS)
- Disease-free Survival (DFS)
- Therapy-associated toxicity/morbidity
  - ◦ During therapy (acute toxicities)
  - ◦ late morbidity 0.5, 1, 2 and 3 years after end of therapy
- Swallowing function (using MD Anderson dysphagia inventory)
- Quality of Life (QoL, using EORTC QLQ-C30 an QLQ-H&N43)
- Incremental cost-effectiveness ratio (ICER) in costs/QALY
- Direct and indirect costs at 3 years after randomization

#### Inclusion criteria

- Histologically proven SCC of the oropharynx; T1, N2a-c, M0; T2, N1–2c, M0; T3, N0-2c, M0, amenable to transoral resection)
- Primary tumor must be resectable through transoral approach
- p16 immunohistochemistry by local pathology or FFPE tissue must be available for central HPV diagnostics
- Written and signed informed consent
- Briefing through surgeon and radiation oncologist
- ECOG PS ≤ 2, Karnofsky PS ≥ 60%
- Age ≥ 18
- Curative treatment intent
- Adequate bone marrow function: leucocytes > 3.0 × 109/L, neutrophils > 1.5 × 109/L, platelets > 80 × 109/L, hemoglobin > 9.5 g/dL
- Adequate liver function tests: Bilirubin < 2.0 g/dL, SGOT, SGPT, < 3 x ULN
- If of childbearing potential, willingness to use effective contraceptive methods for the study duration and 2 months post-dosing.
- dental examination and appropriate dental therapy if needed prior to beginning of radiotherapy
- Nutritional evaluation prior to initiation of therapy and optional prophylactic gastrostomy (PEG) tube placement

#### Exclusion criteria

- Prior invasive malignancy except controlled skin cancer or carcinoma in situ of cervix
- Unknown primary (CUP), nasopharynx, hypopharynx, laryngeal or salivary gland cancer
- Metastatic disease
- Serious comorbidity, e.g. high-grade carotid artery stenosis, congestive heart failure NYHA grade 3 and 4, liver cirrhosis CHILD C
- Hemoglobin level < 9.5 g/dl within 4 weeks before randomization
- Pregnancy or lactation
- Women of child-bearing potential with unclear contraception
- Previous treatment with chemotherapy, radiotherapy, EGFR-targeting agents or surgery exceeding biopsy in head and neck
- Concurrent treatment with other experimental drugs or participation in another clinical trial with any investigational drug within 30 days prior to study screening
- Social situations that limit compliance with study requirements or patients with an unstable condition (e.g., psychiatric disorder, a recent history of drug or alcohol abuse, interfering with study compliance, within 6 months prior to screening) or otherwise thought to be unreliable or incapable of complying with the requirements of the protocol
- Patients institutionalized by official means or court order
- Deficient dental preservation status or not accomplished wound healing

#### Clinical examination and assessments

- Signing of consent
- History and physical examination by a radiation oncologist and head and neck surgeon including panendoscopy
- CT or MRI Neck (according to local routine)
- CT Chest/abdomen (if clinically indicated)
- Performance Status (ECOG/Karnofsky) and ASA classifications
- Vital signs including blood pressure, heart rate, body temperature and electrocardiogram (ECG)
- Laboratory test: hematology panel (hemoglobin, platelets, WBC and WBC differential with neutrophils, lymphocytes, monocytes, eosinophils, basophils) chemistry panel (sodium, potassium, calcium, creatinine, total and direct bilirubin, alkaline phosphatase, ALT, AST, CrP, INR, PTT). HIV and β-HCG only at baseline.
- Audiometry including audiogram
- EQ-5D-5L version 2.0, QLQ-C30 version 3.0: Questionnaires to assess health related quality of life; QLQ-H&N43 version 1.0
- Reporting for adverse events according to CTCAE/RTOG
- MD Anderson dysphagia inventory (MDADI)
- Reporting for late morbidity according to CTCAE/RTOG

### Treatment plan

Both treatment arms represent state of the art procedures for the treatment of transorally resectable oropharyngeal cancer. Staging procedures are identical for both treatment arms.

### Arm A

#### Transoral surgery

During the initial panendoscopy and first assessment (EUA, examination under anesthesia), the transoral accessibility/exposure of the tumor will be assessed by the same attending surgeon who will perform the definitive tumor resection as well (inclusion criteria).

Definitive surgery should generally be performed within 2 weeks, but not more than 4 weeks after randomization. The appropriately indicated neck dissection(s) may be performed either prior to, during the same session, or within 2 weeks after the resection of the primary tumor, but not later than 4 weeks following randomization. The primary tumor must be resected with clear margins (R0) and en bloc in all cases. Frozen section assessment must be routinely and readily available.

The specifically and appropriately chosen surgical technique/modality for transoral resection will be determined at the discretion of the attending head and neck surgeon. Further to conventional surgical cutting tools (energy as well as cold steel surgical instruments), TOLM/TLM (transoral laser microsurgery) and TORS (transoral robotic surgery) are commonly the default choices. Laser-powered TORS is also a possibility, using a hollow conduit drop-in guide with the robotic endowrist instruments.

A “clear margin” is defined as ≥5 mm distance from the invasive tumor front to the resection margin. If the surgeon obtains additional margins from the tumor site, the “new” margins should be referred to the geometric (3D) orientation of the resected main tumor specimen. A statement by the pathologist in the final histopathology report should point out that this “new” margin represents the final margin of the resection, in addition to the histological status of the main specimen.

A “close margin” is defined as < 5 mm distance from the invasive tumor front to the resected margin that is still not involved (R0). Statements in the final histopathology reporting less than 2 mm as the closest margins are considered as involved margins (R1).

Reconstruction of the surgical defect may be performed at the discretion of the attending surgeon, using any kind of flaps, i.e. free, pedicled or local/regional flaps. Primary closure or healing by secondary intention is recommended when regarded as functionally appropriate, but this attempt must not compromise obtaining wide, tumor-free margins.

### Neck dissection

In the surgical study arm, appropriately indicated selective (SND) or modified radical neck dissections (mRND) are included by default, according to the tumor staging (N-classification) and localization of the primary tumor.

By default, patients will undergo neck dissection including at least Levels II, III and IV in all cases. Further levels, e.g. level I and/or V will be included if they are involved. N-positive necks may still be treated with selective neck dissection, especially in the absence of clinically obvious macroscopic extracapsular spread (ECS).

Patients will receive ipsilateral SND levels II-IV or mRND for well-lateralized lesions of the soft palate, tongue-base, tonsillar region and/or glossopharyngeal sulcus, as well as for posterior pharyngeal wall tumors not approaching the midline within 1 cm. For all other sites and expansions, bilateral neck dissections will be performed.

The overall nodal yield should include at least 15 lymph nodes, irrespective of their level or their metastatic involvement. A minimum nodal yield of 15 lymph nodes per dissected side of the neck is recommended [24] and is subject to quality assurance review. Removal of < 15 lymph nodes will be considered as minor protocol deviation and recorded.

Lymph node levels must be divided and clearly marked by the attending surgeon on site, before handing them over to pathology to assign each harvested lymph node to its level of origin. This is of paramount importance for the planning of adjuvant radiation therapy, if applicable, and for quality control purposes.

### Adjuvant (chemo-)radiotherapy (CRTX)

Adjuvant and definitive(C) RTX protocols of this study are currently internationally accepted standard of care procedures. According to local routine, radiotherapy protocols as listed below should be used. Intensity modulated radiotherapy (IMRT) will be used for all patients in this study.

Standard adjuvant treatment protocols, if indicated and applicable, must begin within 6 weeks (42 days) post-surgery in Arm A.

### Localization, simulation and immobilization

All patients will be placed in a supine position and immobilized using a thermoplastic mask prior to CT simulation (slice thickness of 2–3 mm) with intravenous contrast media (100 ml, e.g.Ultravist®), if feasible.

### Target volume definition and selection

#### Clinical target volume (CTV) definition

Fusion of initial tumor images to the planning CT scan must be performed routinely. Clinical information and description of findings in panendoscopy and surgical reports as well as the pathohistological report should be used for target definition. In general, the volume definition of cervical lymph node levels should be performed using the RTOG head and neck lymph node atlas (www.rtog.org) and additional recommendations for delineation and selection of elective neck levels [25, 26].

##### Arm A (surgery+adjuvant RT)

The CTV1 consists of a 1–1.5 cm anatomically expansion (e.g. retracted from air and bone) of the initial gross tumor volume (GTV)/ and the tumor bed (Intermediate-risk (IR): primary tumor, involved lymph nodes; High-risk (HR): primary tumor with R1 resection, lymph node metastasis with ECS) [27, 28].

The CTV2 includes for IR- and HR-patients the subclinical (elective) lymph node levels, and for HR patients the lymph node levels with lymph node metastasis without ECS, and 1–1.5 cm expansion of the initial gross tumor volume (GTV)/and the tumor bed of primary side with clear margins.

##### Arm B (definitive RT)

The CTV1 consists of the GTV of the primary and the macroscopically involved lymph nodes (GTV1) with 0–15 mm margin (volumetric expansion) [29] while the CTV2 includes the soft tissue within a 10 mm margin of the CTV1.

CTV3: Elective nodal levels at risk.

#### Planning target volume (PTV) definition

##### Arms A and B

The PTV consists of the tumor bed with involved resection margins - R1 - and/or regions of ECS.

PTV should not go outside the skin surface, and can be retracted from the skin surface by 2 mm. If daily image-guided radiotherapy (IGRT) is not used, the minimum CTV-to-PTV margin should be 5 mm; in general, it should not exceed 10 mm for significant inter-fraction variability such as tongue. Institutions using daily IGRT for margin reduction, the minimum CTV-to-PTV margin should be 3 mm; it should not exceed 5 mm for significant inter-fraction variability such as tongue.

### Radiotherapy planning

Static or rotational IMRT using megavoltage photon beams is mandatory for this trial.

All plans must be normalized such that 95% of the volume of the PTV1 is covered with the prescribed dose according to ICRU report 83 (median prescribed dose to 100% PTV for SIB to PTV1, D_min_ 95%, D_max_ 107%). At 1 cc PTV1 volume on the DVH curve, the dose should not be > 110% of the prescribed dose. At a volume of 0.03 cc within the PTV1 volume on the DVH curve, the dose should not be < 95% of the prescribed dose. For any volume of tissue outside the PTVs that has a size of 1 cc, the dose should not be > 107%.

### Adjuvant radiotherapy and chemotherapy (CRTX)

Adjuvant CRTX will be administered according to the RTOG and EORTC high-risk criteria published in 2004 [30].

High-risk patients (HR) are defined as patients with

- ECS
- positive surgical margins on final surgical histopathology report (R1)

Simultaneous-integrated boost IMRT *(*SIB) may be delivered in 30 fractions with 5 × 1.8 Gy/week to a total dose (TD) of 54 Gy to the elective planning target volume (PTV2; regions at risk for microscopic disease) and 5 × 2.13 Gy to a TD of 63.9 Gy to the PTV1 (tumor bed with involved resection margins - R1 - and/or regions of ECS) with appropriate margins.

Alternatively, SIB may be delivered in 33 fractions with 5 × 1.8 Gy to a TD of 59.4 Gy to the PTV2 and 5 × 2 Gy to the PTV1 (R1, ECS positive) to a TD of 66Gy.

According to local routine, intravenously (i.v.) applied chemotherapy protocols with Cisplatin, Mitomycin C (MMC) or 5- Fluorouracil (5-FU) listed below in Table 1 should be used. They are currently internationally accepted standard of care procedures. Subsequent chemotherapy doses should follow the protocol specified days of treatment plus/minus 2 days.

**Table 1:** chemotherapy protocols

Option	Treatment	Days of treatment	Annotation
**1**	Cisplatin 100 mg/m^2^ i.v.	Days 1, 22 and 43	
**2**	Cisplatin 30 − 40 mg/m^2^ i.v.	Weekly (days 1, 8, 15, 22, 29 and 36)	
**3**	Mitomycin C 10 mg/m^2^ i.v. 5-FU 600 mg/m^2^/day i.v.	Days 1, 29 Days 1–5	Only in case of GFR < 60 ml/min
**4**	Cisplatin 20 mg/m^2^ i.v. 5-FU 600 mg/m^2^/day i.v.	Days 1–5 and 29–33 Days 1–5 and 29–33	

Adjuvant radiotherapy alone is recommend for patients with intermediate-risk factors (IR):

- multiple positive lymph nodes
- “close margins” (less than 5 and more than 2 mm)
- lymphatic/vascular/perineural invasion
- pT3 primary
- positive level IV or V nodes

These patients will receive adjuvant radiotherapy only, by means of static or rotational IMRT. SIB may be delivered in 25 fractions with 5 × 2 Gy/week to a TD of 50 Gy to the elective PTV2 (regions at risk for microscopic disease), and 5 × 2.24 Gy to TD 56 Gy to the PTV1 (tumor bed with margins).

Alternatively, SIB may be delivered in 30 fractions with 5 × 1.8 Gy to a TD of 54 Gy to the PTV2 and 5 × 2 Gy to the PTV1 to a TD of 60Gy. Irradiation of the contralateral neck is controversial and will left to the discretion of the treating radiation oncologist.

It will be possible in some cases that adjuvant therapy is not necessary. Some necks may be “downstaged” after pathological staging from cN2a to pN1 or pN0. With negative margins and no perineural or lymphovascular invasion, these patients would not require post-operative RT but will be kept in the study and followed according to protocol.

### Arm B: definitive radiotherapy

#### Radiotherapy

Protocol treatment must begin within 4 weeks (28 days) post randomization for Arm B.

For the static or rotational IMRT one of the following schedules should be used:

1. SIB in 32 fractions in 5 × 1.7 Gy to a TD of 54.4 Gy to PTV3 (elective neck levels), 1.9 Gy to a TD of 60.8 Gy to PTV2 (GTV + 1 cm CTV, and ≥ 5 mm PTV margin) and 2.2 Gy to a TD of 70.4 Gy to the PTV 1 (GTV Tumor and GTV lymph nodes plus isotropic ≥5 mm PTV).
2. Sequential boost IMRT will be delivered in 35–36 fractions over 6–7 weeks, 5 weekly fractions of 2 Gy (TD to PTV1: 70–72 Gy; TD to PTV2: 60Gy; TD to PTV3: 50 Gy).
3. Hyperfractionated accelerated radiation therapy (HART using 2 Gy/ fraction (5 times per week) up to 30 Gy; followed by 1.4 Gy/fraction twice-a-day radiation therapy (BID) to a total dose of 49.6 Gy (low risk subclinical disease). High-risk subclinical sites will then be taken to 59.4 Gy with 1.4 Gy/ fraction BID followed by a boost to the primary tumor and involved nodes (CTV1 consists of a 0.5–1.5 cm expansion of the gross tumor volume; GTV) to cover potential local invasion to a cumulative dose of 72 Gy at 1.4 Gy BID.

### Salvage surgery in Arm B

In Arm B, the first radiological assessment should be performed 6 weeks after end of treatment. Post-treatment residual primary tumor or neck nodes may be subject to salvage surgery within 8–12 weeks after completion of radiation treatment. Positive primary and/or neck specimens will be considered as local and/or locoregional failures.

### Quality assurance

An independent data-monitoring committee (IDMC) will follow the progress of the clinical trial, evaluate enrolment, safety, data quality, and primary efficacy parameters and will propose changes, ending or continuing of the trial to the sponsor.

The monitoring will be conducted in compliance with ICH-GCP and according to the monitoring plan and will be performed by the CTC North GmbH & Co. KG, Hamburg.

Criteria for assessing efficacy and safety endpoints will be standardized by using NCI-CTCAE version 4.03 and according to RTOG acute and late radiation morbidity for safety issues and RECIST version 1.1 for efficacy parameters (to determine disease recurrence). Every center has to reveal their laboratory norm values and their validation through certification.

To ensure quality of data, study integrity, and compliance with the protocol and the various applicable regulations and guidelines, the sponsor may conduct site visits to institutions participating to protocols.

### Visit schedule, follow-up and assessment of efficacy

The visit schedule (Table 2) consists of a baseline visit within 4 weeks prior to randomization for both arms.

**Table 2:** Visite schedule

	Baseline	Randomization	Treatment phase (start within 4 weeks after randomization)				
Treatment Arm			*Arm A (8–17 weeks)*			*Arm B (6–11 weeks)*	
			Postoperative Visit	Intermediate Visit^i^	Final Visit	Intermediate Visit^i^	Final Visit
**Study week (W)**	−4	0	1–4	9–13	12–17	3–7	6–11
**Informed consent**^b^**(Study and translational research)**	-4						
**Medical history incl. smoking and alcohol, demographics**	X						
**Eligibility criteria**^a^	X						
**CT or MRI Neck**	X						
**CT Chest/Abdomen**	X						
**Physical examination**^**b**^	−2		X	X	X	X	X
**Performance status/ASA**	−2		X	X	X	X	X
**Vital signs**^**c**^	−2		X	X	X	X	X
**Laboratory determinations**^**d**^	X		X	X	X	X	X
**Panendoscopy, FFPE tissue**^a^	X		X^e^				
**Audiometry**	X				X		X
**Blood draw translat. research**^a^	X		X	X^j^	X	X^j^	X
**Dental evaluation and panoramic view as indicated**	X						
**Postoperative morbidity**^a^	X		X	X	X		
**Swallowing function**^a f^	X		X	X	X	X	X
**Nutritional evaluation**	X						
**Quality of life assessment**^a **g**^	X		X	X	X	X	X
**Health Care Utilization and Productivity loss**^a **h**^	X						
**Monitoring AE’s/SAE’s**^a^			X (Randomization to 28 days after the last administration of IMP and/or 5 months after randomization in this trial)				
**Survival**			X				

^a^Study specific procedures

^b^including weight, height (only baseline)

^c^blood pressure, heart rate, body temperature. ECG at baseline and as clinically indicated

^d^haematology panel (haemoglobin, platelets, WBC with neutrophils, lymphocytes, monocytes, eosinophils, and basophils), chemistry panel (sodium, potassium, calcium, serum creatinine, alkaline phosphatase, AST, ALT, total and direct bilirubin, CrP; glomerular filtration rate by MDRD, coagulation (INR, aPTT, PT). HIV and ß-HCG only at baseline and as clinically indicated

^e^surgical resected tumor specimen

^f^MDADI Score (Appendix E)

^g^Quality of life assessments using EQ-5D-5L, EORTC QLQ-C30, QLQ H&N-43 (Appendix D)

^h^Self-report inventory based on FIMA (Appendix A) and sociodemographic evaluation (Appendix I)

^i^3 weeks after start of CRTX

^j^within 2 weeks after end of CRTX

Staging should be completed at this point including head and neck exam, biopsy taken during panendoscopy under general anesthesia, ultrasonography of the neck, MRI (recommended) or CT scan with intravenous contrast of primary and neck and exclusion of distant metastasis according to local routine including CT of the chest and at least ultrasonography of the upper abdomen within 4 weeks prior to randomization.

Treatment must start within 4 weeks after randomization.

Arm A includes 3 visits while Arm B consists of 2 visits: The postoperative visit in Arm A should be conducted 2 weeks postoperatively and as clinically indicated. Intermediate visit should be performed after 3 weeks of CRTX start in both arms. The final visit should be conducted at the end of radiotherapy in both arms.

At each visit, physical examination, performance status, vital signs and labs should be conducted as clinically indicated and according to local routine. Relevant surgical adverse events and non-surgical related adverse events (cardiovascular, pulmonary, renal and others) as well as death (including cause of death) must be recorded and documented in eCRF.

The postoperative visit in Arm A and the intermediate visit in both arms include quality of life, swallowing function, health care utilization and productivity loss assessments.

Additionally, quality of life and swallowing function should be conducted at the final visit.

Follow-up will be performed until end of study, which means 36 months after end of treatment of the last patient.

First follow-up visit to assess locoregional tumor control will be performed after 6 weeks. In Arm B, the first radiological assessment should also be performed at this time. In case of suspicious presence of persistent disease, a second imaging study should be performed within 12 weeks after end of therapy. If there is persistent suspicious residual disease at the primary site, panendoscopy including tumor biopsy should be performed to confirm or exclude residual tumor tissue. In case of residual tumor, the treating clinicians will determine the treatment of residual disease at the primary site. If the primary site is cleared of disease, and residual disease in the neck is suspected on imaging and/or clinical evaluation, a salvage neck dissection will be performed within 12 ± 2 weeks of completion of IMRT. Positive histology of the primary site will be considered as local failure and positive neck specimens will be considered as locoregional failure.

In the further course all subjects will be followed every 3 months (+/− 30 days) for at least 36 months and until end of study. After 36 months patients should be followed up every 6 months until end of study, according to local routine. End of study will be 36 months after end of treatment of the last patient.

Evaluation for disease recurrence will be performed by clinical examination including:

- Physical examination including complete head and neck exam, ultrasonography of the neck, weight, vital signs, performance status (ECOG/ Karnofsky), assessment of toxicity
- Quality of life assessment
- Assessment of health care utilization and productivity loss
- Swallowing function
- Disease assessment according to RECIST v1.1
  - ◦ contrast enhanced MRI (CT) of the neck at first follow up, month 6, 18, 30 and in case of suspicion of recurrence
  - ◦ CT chest month 12, 24, 36
  - ◦ In case of unclear or suspicious lesion in thorax or abdomen: CT chest, CT/ultrasound abdomen.

### Measurement of response

Time to local or locoregional failure (LRF) will be recorded as time from randomization to date of first observed treatment failure confirmed by histologically proven tumor persistence or recurrence (either locally or locoregionally) or death from any cause.

Overall survival will be determined as time from randomization to date of death from any cause.

Disease-free survival (DFS) will be recorded as time from randomization to date of first observed disease recurrence (either local, locoregional or distant) or death from any cause. Second malignancy will not be counted as event in the DFS analysis.

Diagnosis of recurrence could either be made by radiological imaging or by positive cytology or biopsy.

All radiological tumor assessments will be collected and retrospectively reviewed for pattern of recurrence and locoregional control.

Tumor measurement and disease assessment will be performed according to RECIST v1.1. Follow-up will be compared to baseline assessments and response criteria are defined as follows:

- Complete Response (CR): Disappearance of all target lesions.
- Partial Response (PR): At least a 30% decrease in the sum of diameters of target lesions, taking as reference the baseline sum of diameters.
- Progressive Disease (PD): At least a 20% increase in the sum of diameters to target lesions, taking as reference the smallest sum on study (this may include the baseline sum). The sum must also demonstrate an absolute increase of at least 5 mm.
- Stable Disease (SD): Neither sufficient shrinkage to qualify for PR nor sufficient increase to qualify for PD.
- Additionally, volumetric measurements will be performed from every MRI and CT using OsirixTM (FDA approved version, 64-bit) by the radiologic reference center.

### Statistics and sample size calculation

Two hundred eighty patients will be randomized to one of the two arms of the study with an allocation ratio of 1:1. Treatment allocation will be performed centrally by eCRF.

### Sample size calculations

The trial is based on an event-driven design with a planned observational period of 5 years (recruitment time 2 years and follow-up time 3 years).

The event rate in the definitive chemoradiotherapy for oropharyngeal cancer group is assumed to be 50% after 36 months. The transoral head and neck surgery followed by adjuvant (chemo) radiotherapy is assumed to reduce the event rate for the primary outcome to 35%. It is assumed that the hazard rate is constant over time. Under these assumptions, 142 events have to be observed during the planned observation period, which will result in a sample size of 280 patients.

In both arms a 3% lost to follow-up during the study is estimated. After recruiting 250 patients a blinded interim analysis will be performed. The steering committee will decide on adaptation of the sample size/ recruiting time. Additionally, based on results of the planned unblinded interim analysis after 50 and 75% of available observed events, the steering committee will decide on adaptation of the recruiting/follow up time or to allow an early stopping of the trial for success. The recruitment will be stopped immediately if the needed number of events is reached.

### Statistical analysis

The primary analysis is in the intention-to-treat population (ITT), consisting of all randomized patients.

Analysis of time to event will be done with Cox regression and Kaplan-Meier curves for both arms, adjusted to the group sequential design in a way that a two-sided overall significance level of 5% is kept. Descriptive statistics will be measured for all patients and separately for both arms.

### Interim analysis

It may be required to adapt sample size and recruiting time, to reach the required number of events. This may lead to an increased average follow-up time.

A group sequential design was chosen with two unblinded interim analyses after 50 and 75% of the required events have been observed. At each interim analysis, early stopping for efficacy is allowed according to the rules resulting from the sample size calculation. In order to preserve the blindness of the investigators including the steering committee, at the two interim analyses, the statistician of the independent data monitoring committee (IDMC) will link the time-to-event data collected so far to the randomization code, calculate the log-rank statistic and compare it to the predefined limits.

### Accompanying scientific support programs

#### Translational research establishing biomarkers predicting outcome of primary surgery as well as definitive chemoradiation

Apart from HPV and the HPV-surrogate marker p16, established prognostic markers for oropharyngeal cancer are rare. Therefore, within the current trial, tumor tissue and blood will be collected together with the clinical data. Besides the clinical case report form, a central database will be established at the coordinating study site, gathering the data of the available patient samples to enable translational research.

The translational program of the study will focus on the contribution of main components of the most relevant cellular functional pathways determining failure of treatment in OPSCC. Factors from various functional circuits that may prominently contribute to the resistance to chemoradiotherapy (CRTX) will be analyzed and assessed for their potential to predict local and locoregional failure in both treatment arms. The data acquired will be used to establish biomarkers indicating the need for an initial surgical intervention.

Tumor tissue will be analyzed using immunohistochemistry (IHC), FISH staining and mutational analysis. This will include candidate markers involved in tumor cell signaling, DNA-repair, immune modulation as well as potential cancer stem cell markers and markers of proliferation, vascularization, and hypoxia.

It is also planned to use patient blood samples to characterize circulating tumor cells (CTC) as a predictor for distant metastasis and, as recently suggested, for local recurrence as well [31, 32]. In case of HPV-positive tumors, blood samples will also be used to assess anti-E6, anti-E7 and anti-L1 immune responses.

In addition to the above, further markers, which might gain importance during the course of the trial, will also be analyzed. It is the aim of the organizers to incorporate all relevant groups in Germany working in these fields to cover all aspects.

#### Predictive value of volumetric measurement and DWI-MRI in oropharyngeal cancer

Besides the evaluation criteria according the guidelines for **R**esponse **E**valuation **C**riteria **i**n **S**olid **T**umors (RECIST, Version 1.1), an additional volumetric assessment of the primary tumor site might have further prognostic value. RECIST 1.1 is based on unidimensional lesion measurement for the overall evaluation of tumor burden. This raises the question whether volumetric anatomical assessment or functional assessment, like diffusion-weighted imaging (DWI) for magnetic resonance imaging (MRI), may obtain advantages over anatomical unidimensional assessment. DWI is an emerging MRI technique for response prediction in HNSCC patients treated with CRTX [33]. DWI is based on the differences in water mobility in different tissue types, which can be quantified into an apparent diffusion coefficient (ADC). Higher pretreatment ADC values are associated with adverse prognosis [34–36]. Furthermore, DWI has shown potential to detect central necrosis and micrometastatic lymph nodes [37].

Volumetric assessment and DWI will be performed in both arms, to retrospectively appraise tumor volume as a possible predictive factor for therapy response. In addition, a correlation analysis between the maximal diameter of the tumor and the volumetric measurement of the relevant oropharyngeal region will be performed.

## Discussion

For loco-regionally advanced, but transorally resectable OPSCC, the current standard of care includes surgical resection and risk-adapted adjuvant (chemo) radiotherapy, or definite chemoradiotherapy with or without salvage neck dissection. The choice of first-line treatment is subject to regional and cultural preferences. For instance, in the U.S., in the Netherlands, in Denmark or in France, OPSCC is predominantly treated by concurrent CRTX, while in Germany first-line treatment is traditionally dominated by surgery [38].

Each one of these strategies has already been extensively investigated and verified in prospective trials, although the majority of the studies focused on the conservative modalities [30, 39–45]. In contrast to the Anglo-Saxon countries, transoral surgical approaches have been used frequently in Germany to treat patients with oro-, hypopharyngeal and laryngeal cancer [16]. However, only a few multicenter studies and no prospective controlled trials have been performed to date [17, 18].

To shed light on this topic first efforts were made by the RTOG (Radiation Therapy Oncology Group). RTOG 1221 was a phase IIb trial which compared transoral surgery and neck dissection followed by risk-adapted adjuvant therapy to definitive chemoradiation in HPV-negative oropharyngeal cancer (NCT01953952). Unfortunately, this trial was closed early due to lack of accrual (no patient in 15 months); thus, no results became available. Possible reasons were a paucity of eligible p16-negative patients with stage T1–2, N1–2b OPSCC and concerns of a few physicians and patients for whom random assignment of up-front therapy might have been difficult to implement. Moreover another prospective surgical trial with similar inclusion criteria was open at the same time (ECOG 3113) [46].

In comparison to the TopROC trial the RTOG 1221 trial was designed as prospective, randomized controlled trial with the surgical approach as experimental arm. Due to the extensive experience in Germany for transoral surgery, a randomized controlled study to compare standard therapy (chemoradiation) with experimental therapy (transoral surgery) was not reasonable. Therefore the TopROC-trial was designed as comparative effectiveness trial to measure the effectiveness of the compared treatments and to realistically reflect the implemented procedures in our health care system. Furthermore, the informed consent discussion will be performed with the patient, surgeon and radiotherapist together to improve the inclusion of patients.

Recently, results of a study randomizing between surgery and radiotherapy were presented to the community the first time [21]. The ORATOR-trial is a phase II study comparing radiotherapy (arm 1) to TORS (arm 2) in early-stage OPSCC (T1/T2). Sixty-eight patients were randomly assigned to arm 1 or arm 2. The arms were well balanced concerning baseline factors like gender, smoking history, primary tumor site, clinical stage, baseline scan and p16-status. The primary endpoint was QoL 1-year post-treatment assessed with the MDADI. Secondary endpoints were overall survival, progression-free survival, QoL at other time points (MDADI, EORTC QLQ-C30 and H&N35 scales, Voice Handicap Index, Neck Dissection Impairment Index and the Patient Neurotoxicity Questionnaire), toxicity and swallowing function (measured by feeding tube rate at 1-year, MDADI and CTC-AE Dysphagia scores).

The median follow-up time was 27 months (arm 1) and 29 months (arm 2). The MDADI score at year 1 differed between the arms. A 10-points change was considered as a clinically meaningful change. This endpoint was not reached in the ORATOR-trial as the change was only 6.8 points. Only the emotional and the functional subscale reached a clinically meaningful decline at year 1. The changes in MDADI scores between the two groups over the time showed no statistically significance concerning the MDADI global score. The changes were statistically significant concerning the MDADI subscales but with average differences below the threshold of a clinically meaningful change.

The spectrum of toxicity differed between the arms. Patients undergoing surgery showed less tinnitus, hearing loss, neutropenia, and constipation than patients undergoing radiotherapy. Overall and the progression-free survival were excellent in both groups with no significant difference. However, the study was not designed to compare survival outcomes. The authors concluded that the clinically-similar QoL outcomes and the differences in toxicities are indicating a shared decision making between clinicians and patients and that patients with OPSCC should be informed about both treatment options.

In comparison to the ORATOR trial 280 patients with locally advanced but transorally resectable oropharyngeal OPSCC (T1, N2a-c, M0; T2, N1–2c, M0; T3, N0-2c, M0) will be randomized in the TopROC trial. The study is powered to evaluate the time to local or locoregional failure. Secondary endpoints are overall and disease-free survival, therapy-associated toxicities/ morbidity, swallowing function and QoL. The aim of this trial is to evaluate the effectiveness of primary surgical versus non-surgical treatment of locally advanced, but transorally resectable oropharyngeal cancer in terms of time to local or locoregional failure or death from any cause (LRF).

Both therapies are accepted internationally as standard of care alternatives. In clinical practice, the surgical or conservative approach is chosen primarily based on the experience and preferences of the individually treating physician. Internationally, primary chemoradiation is the standard treatment for oropharyngeal cancer. In Germany, however, primary tumor resection with adjuvant radio-(chemo-) therapy has established itself as a standard therapy. The key question is therefore whether one of these therapeutic approaches is more effective in clinical routine, i.e. under the real conditions of our health care system and without selection for ideal patients. We currently assume that the surgical approach is superior in terms of local and locoregional control.

Especially considering the fact that the incidence of HPV-positive OPSCC is increasing over the last decades [47] there is a high need to improve criteria for treatment decision and QoL after cancer treatment for these mainly younger and healthier patient cohort. It is a burning issue to provide level Ia evidence for this specific treatment setting in terms of a randomized clinical trial. Choosing a study design in terms of a comparative effectiveness study aims to reflect the real clinical daily routine. The mandatory medical need in this field was also recognized by the German Cancer Aid. This study is funded by the German Cancer Aid (Deutsche Krebshilfe, project number 1120027) and started recruitment in 2018.

## Supplementary information

**Additional file 1.**

**Additional file 2.**

**Additional file 3.**

**Additional file 4.**

## Data Availability

The datasets generated and/or analysed during the current study are not publicly available due ongoing trial in process but are available from the corresponding author on reasonable request.

## Abbreviations

ADC: Apparent diffusion coefficient
AE: Adverse event
ANC: Absolute neutrophil count
ALT (SGPT): Alanine aminotransferase (serum glutamic-pyruvic transaminase)
AST (SGOT): Aspartate aminotransferase (serum glutamic-oxaloacetic transaminase)
eCRF: Electronic case report form
CrP: C reactive protein
CRTX: Chemoradiotherapy / chemoradiation
CT: Computerized tomography
CTC: Circulating tumor cells
CTCAE: Common terminology criteria for adverse events
CTV: Clinical target volume
DFS: Disease free survival
DVH: Dose-volume Histogram
DWI: Diffusion-weighted imaging
ECS: Extracapsular spread
ECG: Electrocardiogram
ECOG: Eastern cooperative oncology group
ENT: Ear, nose, throat department
EORTC: European organisation for research and treatment of cancer
ESMO: European society of medical oncology
EUA: Examination under anaesthesia
FDA: Food and drug administration (U.S. government agency)
FFPE: Formalin-fixed, paraffin-embedded (tissue)
FISH: Fluorescence in-situ hybridization
GCP: Good clinical practice
GTV: Gross tumor volume
Gy: Gray
HNSCC: Head and neck squamous cell carcinoma
HPV: Human papillomavirus
HR: High-risk
ICER: Incremental cost-effectiveness ratio
IDMC: Independent data monitoring committee
IGRT: Image-guided radiotherapy
IHC: Immunohistochemistry
IMRT: Intensity modulated radiotherapy
INR: International normalized ratio
ITT: Intention-to-treat population
iv: Intravenous
LRF: Time to local or locoregional failure
MDADI: M.D. Anderson dysphagia inventory
mRND: Modified radical neck dissection
MRI: Magnetic resonance imaging
NCCN: National comprehensive cancer network
NCI: National cancer institute
NYHA: New York heart association
OPSCC: Oropharyngeal squamous cell carcinoma
OS: Overall survival
PFS: Progression-free survival
PTT: Partial thromboplastin time
PTV: Planning target volume
QALY: Quality adjusted life-years
QLQ: Quality of life questionnaires
QoL: Quality of life
RECIST: Response evaluation criteria in solid tumors
RTOG: Radiotherapy oncology group
SAE: Serious adverse event
SCC: Squamous cell carcinoma
SD: Stable disease
SIB: Simultaneous integrated boost
TD: Total dose
TLM: Transoral laser microsurgery
TORS: Transoral robotic surgery
ULN: Upper limit of normal
WBC: White blood cell count
