# Supplementary material for: Comparative effectiveness trial of transoral head and neck surgery followed by adjuvant radio(chemo)therapy versus primary radiochemotherapy for oropharyngeal cancer (TopROC)
Source: BMC Cancer. 2020 Jul 29;20:701. doi: 10.1186/s12885-020-07127-2 (PMC7389683; doi:10.1186/s12885-020-07127-2)
Supplement: Supplementary file 1 — Additional file 1. [file 12885_2020_7127_MOESM1_ESM.pdf]

Visite (z.B Baseline):

Datum:

Pat.ID:

FIM_HNonko©

Fragebogen zur **I**nanspruchnahme **m**edizinischer und nicht-medizinischer Versorgungsleistungen bei onkologischen HNO-Patienten

Bitte beginnen Sie auf der nächsten Seite mit dem Ausfüllen des Fragebogens.

Vielen Dank für Ihre Unterstützung!

© Urheberrechtlich geschützt, Version 1.0 vom 23.02.15, Institut für Gesundheitsökonomie und Versorgungsforschung (IGV)

In dem folgenden Fragebogen werden zwei verschiedene Zeiträume abgefragt (7 Tage und 3 Monate). Bitte achten Sie bei den jeweiligen Fragen, auf welchen Zeitraum sich diese beziehen, und nehmen Sie ggf. bitte einen Kalender zu Hilfe, um die zurückliegende Zeit zu bestimmen.

1) Haben Sie in den **letzten 3 Monaten** einen der folgenden Ärzte aufgesucht? Gemeint ist jeder Besuch in der Arztpraxis, auch wenn der Arzt selbst nicht gesprochen wurde (zum Beispiel Rezept abholen, Blutabnahme). Hausbesuche zählen auch dazu.
Bitte kreuzen Sie zunächst an, ob Sie den jeweiligen Arzt aufgesucht haben (Ja oder Nein). Wenn ja, dann geben Sie bitte weiterhin an, wie oft Sie diesen Arzt aufgesucht haben.

Arzt	Nein	Ja	Wie oft?
Allgemeinmediziner, Hausarzt oder hausärztlicher Internist	☐	☐	☐☐
Psychotherapeut, Psychologe, Psychoonkologe	☐	☐	☐☐
Neurologe / Psychiater	☐	☐	☐☐
Kardiologe (Herzspezialist)	☐	☐	☐☐
sonstiger Fachärztlicher Internist (z. B. Gastroenterologe, Nephrologe, Diabetologe, usw.)	☐	☐	☐☐
Ambulante Behandlung im Krankenhaus zur Notfallversorgung	☐	☐	☐☐
ärztlicher Bereitschaftsdienst, Notarzt	☐	☐	☐☐
Sonstige: ____________________	☐	☐	☐☐

2) Haben Sie in den **letzten 3 Monaten** eines der folgenden therapeutischen Angebote in Anspruch genommen? Hausbesuche zählen auch dazu.
Bitte kreuzen Sie zunächst an, ob Sie die jeweiligen Angebote wahrgenommen haben (Ja oder Nein). Wenn ja, dann geben Sie bitte weiterhin an, wie oft Sie diese Angebote wahrgenommen haben.

Therapeutisches Angebot	Nein	Ja	Wie oft?
Atemtherapie	☐	☐	☐☐
Krankengymnastik (auch Physiotherapie, Massagen, Wärme-, Kältebehandlungen, Stromtherapie oder Heilbäder)	☐	☐	☐☐
Schluck- oder Sprechtherapie	☐	☐	☐☐

3) Haben Sie in den **letzten 3 Monaten** aufgrund Ihres Gesundheitszustandes einen ambulanten Pflegedienst in Anspruch genommen?
Bitte Entsprechendes ankreuzen und ausfüllen

☐ Nein

☐ Ja → Wenn ja, an wie vielen Tagen in der Woche **oder** Tagen in den gesamten 3 Monaten kam der ambulante Dienst zu Ihnen nach Hause?

an ☐ Tagen pro Woche

an ☐☐ Tagen pro 3 Monate

An diesen Tagen, wie lange war der ambulante Dienst bei Ihnen durchschnittlich zu Hause?

durchschnittlich ☐☐ Stunden, ☐☐ Minuten

4) Haben Sie in den **letzten 3 Monaten** aufgrund Ihres Gesundheitszustandes Hilfen von Familienmitgliedern, Freunden, Bekannten oder Nachbarn in Anspruch genommen?
Beispiele für diese Hilfen sind: Hilfe im Haushalt, bei der Körperpflege, beim An- und Auskleiden, bei der Medikamenteneinnahme, beim Einkaufen, bei der Übernahme von Fahrdiensten
Bitte Entsprechendes ankreuzen und ausfüllen

☐ Nein

☐ Ja →

Wenn ja, an wie vielen Tagen in der Woche **oder** Tagen in den gesamten 3 Monaten wurde Ihnen geholfen?

an ☐ Tagen pro Woche

an ☐☐ Tagen pro Monat

An diesen Tagen, wie lange wurde Ihnen durchschnittlich geholfen?

durchschnittlich ☐☐ Stunden, ☐☐ Minuten

5) Erhalten Sie Leistungen aus der gesetzlichen Pflegeversicherung?
Bitte Entsprechendes ankreuzen und ausfüllen

☐ Nein

☐ Ja →

Wenn ja, welche Pflegestufe haben Sie?

Pflegestufe ☐

Falls Ihnen Ihre Pflegestufe nicht bekannt ist: Wie hoch ist das monatliche Pflegegeld?

☐☐☐☐ Euro

6) Haben Sie innerhalb der **letzten 7 Tage** Medikamente verwendet?
Denken Sie bitte auch an Insuline, Hormonersatzpräparate oder länger wirkende Depotmittel.
Bitte Entsprechendes ankreuzen und ausfüllen

☐ Nein

☐ Ja → Wenn Ja, dann lesen Sie bitte aufmerksam nachfolgende Ausfüllhinweise und tragen alle benötigten Angaben zu den verwendeten Medikamenten auf den Seiten 6/7 ein.

Ausfüllhinweise

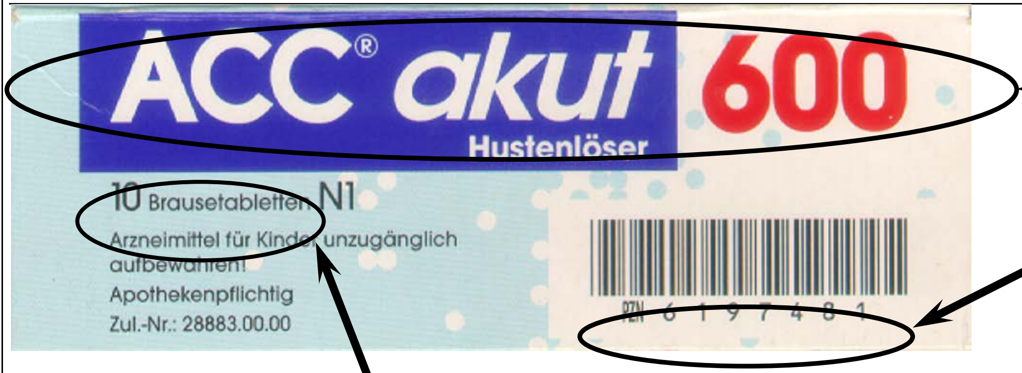

Tragen Sie den **genauen und vollständigen Medikamentennamen** ein.

Tragen Sie die **PZN-Nummer** ein. Sie finden diese meist unter dem **Strichcode** auf einer der Seiten der Medikamentenschachtel. Die Buchstabenfolge „**PZN**" steht immer vor der Nummer; die Nummer ist immer 7- oder 8-stellig. Diese Nummer ist wichtig, damit das Medikament eindeutig bestimmt werden kann.

Tragen Sie die **Packungsgröße** des Medikaments ein:
(zum Beispiel: 10 Brausetabletten, 20 ml, 1 Inhalator, 10 Spritzen, 20 Pflaster)
Tragen Sie die **Dosierung/Menge** mit **Darreichungsform** und **Zeitintervall** ein:
(zum Beispiel: 1 Tablette täglich, 3 mal 20 Einheiten täglich, 1 Spritze pro Woche, alle 3 Tage 1 Pflaster, 3 mal 20 Tropfen täglich, 3 mal 2 Hübe täglich)
Tragen Sie ein, **seit wann** die Einnahme erfolgt:
Monat und Jahr. Falls das Medikament schon länger als 1 Jahr genommen wird, reicht das Jahr.

**Medikamentenname**	**PZN**	**Packungsgröße**<br><br>(Menge in der Packung mit Einheitsbezeichnung)	**Dosierung**<br><br>(Menge pro Tag oder pro Woche/Monat mit Darreichungsform)	**Einnahme seit**<br><br>(Monat/Jahr)
**Beispiel:**<br>***ACC AKUT 600***	|*6*_|*1*_|*9*_|*7*_|*4*_|*8*_|*1*	***10 Brausetabletten***	***3 Tabletten tägl.***<br>***oder***<br>***3 mal 1 Tablette tägl.***	***12/2011***
	|__|__|__|__|__|__|__|			
	|__|__|__|__|__|__|__|			
	|__|__|__|__|__|__|__|			
	|__|__|__|__|__|__|__|			
	|__|__|__|__|__|__|__|			
	|__|__|__|__|__|__|__|			
	|__|__|__|__|__|__|__|			
	|__|__|__|__|__|__|__|			
	|__|__|__|__|__|__|__|			
	|__|__|__|__|__|__|__|			
	|__|__|__|__|__|__|__|			
	|__|__|__|__|__|__|__|			
	|__|__|__|__|__|__|__|			

7) Waren Sie in den **letzten 3 Monaten** zur stationären Behandlung im Krankenhaus?
Bitte Entsprechendes ankreuzen und ausfüllen

☐ Nein

☐ Ja →

Wenn ja, wie oft waren Sie im Krankenhaus?

☐ mal

Fragen zum 1. Krankenhausaufenthalt

Wie viele Tage waren Sie bei Ihrem **1. Aufenthalt insgesamt** im Krankenhaus?

☐☐ Tage

Wie viele Tage lagen Sie **davon** auf einer Intensivstation?

☐☐ Tage

Was war der Grund für Ihren 1. Aufenthalt und wann begann er?
Einweisungsdatum: |__|__|.|__|__|.|__|__|
Grund:___________________

War der 1. Aufenthalt ein geplanter oder ein ungeplanter (Notfall) Aufenthalt?

geplant ☐ ungeplant ☐

Fragen zum 2./3. Krankenhausaufenthalt

Wie viele Tage waren Sie bei Ihrem 2./3. Aufenthalt im Krankenhaus?

☐☐ Tage (2. Aufenthalt) ☐☐ Tage (3. Aufenthalt)

Wie viele Tage lagen Sie **davon** auf einer Intensivstation?

☐☐ Tage (2. Aufenthalt) ☐☐ Tage (3. Aufenthalt)

Was war der Grund für Ihren 2./3. Aufenthalt und wann begann er?
Datum 2. Aufenthalt:|__|__|.|__|__|.|__|__|
Grund:___________________
Datum 3. Aufenthalt:|__|__|.|__|__|.|__|__|
Grund:___________________

War der 2./3. Aufenthalt ein geplanter oder ein ungeplanter (Notfall) Aufenthalt?

2. Aufenthalt		3. Aufenthalt	
geplant	☐	geplant	☐
ungeplant	☐	ungeplant	☐

Falls Sie weitere Krankenhausaufenthalte hatten, bitten wir Sie, diese auf einem zusätzlichen Blatt Papier aufzuschreiben und ebenfalls die Tage insgesamt, die Tage auf der Intensivstation, das Einweisungsdatum, den Grund und die Information, ob es ein geplanter oder ungeplanter Aufenthalt war aufzuschreiben.

8) Waren Sie in den letzten **3 Monaten** zur ambulanten oder stationären Rehabilitation (Kuraufenthalt oder Anschlussheilbehandlung) in einer Rehabilitationseinrichtung?
Bitte Entsprechendes ankreuzen und ausfüllen

☐ Nein

☐ Ja →

Wenn ja, waren Sie zur ambulanten oder stationären Rehabilitation?

Ambulant ☐

Stationär ☐

Wie viele Tage hat die Rehabilitationsbehandlung **insgesamt** gedauert?

☐☐☐ Tage

9) Besitzen Sie eines oder mehrere der folgenden Hilfsmittel?
Bitte kreuzen Sie jeweils an, ob Sie das Hilfsmittel besitzen.
Wenn ja, kreuzen Sie bitte an, ob Sie dieses Hilfsmittel schon länger besitzen oder ob Sie dieses im Rahmen Ihrer Krebstherapie erhalten oder gekauft haben.

Hilfsmittel	Nein	Ja, ich besitze dieses länger	Ja, im Rahmen der Krebstherapie erhalten/gekauft
Rollator	☐	☐	☐
Gehstock	☐	☐	☐
Ernährungspumpe	☐	☐	☐
Absauggerät	☐	☐	☐
Inhalationsgerät	☐	☐	☐
Trachealkanüle	☐	☐	☐
Sonstige ________________	☐	☐	☐

10) Wo wohnen Sie **zurzeit**?

privater Haushalt (keine Seniorenwohnung)	☐
Seniorenwohnung (zum Beispiel: Betreutes Wohnen, Seniorenwohnanlage)	☐
Altenheim	☐
Pflegeheim / Pflegestation im Altenheim	☐

11) Sind Sie berufstätig?
Als Berufstätigkeit zählt auch, falls Sie ein sogenannter „Ein-Euro-Jobber“ sind oder zur Rente etwas hinzuverdienen. Bitte machen Sie in diesem Falle Angaben zur Berufstätigkeit und zur Arbeitslosigkeit/Rente (d. h. mehrere Antworten sind möglich).

- ☐ Ganztags berufstätig
- ☐ Teilzeit berufstätig
- ☐ Arbeitslos (ALG I oder ALG II/Hartz IV) aufgrund meiner Gesundheit
- ☐ Arbeitslos (ALG I oder ALG II/Hartz IV) nicht aufgrund meiner Gesundheit
- ☐ in Rente (vorzeitig aufgrund meiner Gesundheit = Erwerbsminderungsrente, Berufsunfähigkeitsrente)
- ☐ in Rente (nicht aufgrund meiner Gesundheit = Altersrente)
- ☐ Hausfrau/Hausmann (nicht erwerbstätig, nicht berentet, keine Sozialleitungen zum Lebensunterhalt)

12) Falls Sie berufstätig sind, beantworten Sie bitte diese Frage, ansonsten machen Sie bitte weiter mit Frage Nummer 13. Waren Sie während der **letzten 3 Monate** einmal so krank, dass Sie nicht zur Arbeit gehen konnten?

- ☐ Nein
- ☐ Ja →

Wenn ja, wie viele Tage oder Wochen konnten Sie während der letzten **3 Monate** insgesamt wegen Krankheit nicht zur Arbeit gehen?

☐☐ Tage ☐ Wochen

Heutiges Datum ☐☐ ☐☐ 20☐☐
Tag Monat Jahr

Bitte prüfen Sie, ob Sie keine Frage übersehen haben.
Vielen Dank für Ihre Mühe und alles Gute für Ihre Zukunft!
