# Supplementary material for: Comparative effectiveness trial of transoral head and neck surgery followed by adjuvant radio(chemo)therapy versus primary radiochemotherapy for oropharyngeal cancer (TopROC)
Source: BMC Cancer. 2020 Jul 29;20:701. doi: 10.1186/s12885-020-07127-2 (PMC7389683; doi:10.1186/s12885-020-07127-2)
Supplement: Supplementary file 2 — Additional file 2. [file 12885_2020_7127_MOESM2_ESM.pdf]

EORTC QLQ-C30 (Version 3)

Wir sind an einigen Angaben interessiert, die Sie und Ihre Gesundheit betreffen. Bitte beantworten Sie die folgenden Fragen selbst, indem Sie die Zahl einkreisen, die am besten auf Sie zutrifft. Es gibt keine „richtigen“ oder „falschen“ Antworten. Ihre Angaben werden streng vertraulich behandelt.

Bitte tragen Sie Ihre Initialen ein: |__|__|__|__|
Ihr Geburtsdatum (Tag, Monat, Jahr): |__|__|__|__|__|__|__|__|
Das heutige Datum (Tag, Monat, Jahr): 31 |__|__|__|__|__|__|__|__|

	Überhaupt nicht	Wenig	Mäßig	Sehr
1. Bereitet es Ihnen Schwierigkeiten, sich körperlich anzustrengen (z. B. eine schwere Einkaufstasche oder einen Koffer zu tragen)?	1	2	3	4
2. Bereitet es Ihnen Schwierigkeiten, einen <u>längeren</u> Spaziergang zu machen?	1	2	3	4
3. Bereitet es Ihnen Schwierigkeiten, eine <u>kurze</u> Strecke außer Haus zu gehen?	1	2	3	4
4. Müssen Sie tagsüber im Bett liegen oder in einem Sessel sitzen?	1	2	3	4
5. Brauchen Sie Hilfe beim Essen, Anziehen, Waschen oder Benutzen der Toilette?	1	2	3	4

Während der letzten Woche:

	Überhaupt nicht	Wenig	Mäßig	Sehr
6. Waren Sie bei Ihrer Arbeit oder bei anderen tagtäglichen Beschäftigungen eingeschränkt?	1	2	3	4
7. Waren Sie bei Ihren Hobbys oder anderen Freizeitbeschäftigungen eingeschränkt?	1	2	3	4
8. Waren Sie kurzatmig?	1	2	3	4
9. Hatten Sie Schmerzen?	1	2	3	4
10. Mussten Sie sich ausruhen?	1	2	3	4
11. Hatten Sie Schlafstörungen?	1	2	3	4
12. Fühlten Sie sich schwach?	1	2	3	4
13. Hatten Sie Appetitmangel?	1	2	3	4
14. War Ihnen übel?	1	2	3	4
15. Haben Sie erbrochen?	1	2	3	4
16. Hatten Sie Verstopfung?	1	2	3	4

Bitte wenden

**Während der letzten Woche:**	**Überhaupt nicht**	**Wenig**	**Mäßig**	**Sehr**
17. Hatten Sie Durchfall?	1	2	3	4
18. Waren Sie müde?	1	2	3	4
19. Fühlten Sie sich durch Schmerzen in Ihrem alltäglichen Leben beeinträchtigt?	1	2	3	4
20. Hatten Sie Schwierigkeiten, sich auf etwas zu konzentrieren, z. B. auf das Zeitunglesen oder das Fernsehen?	1	2	3	4
21. Fühlten Sie sich angespannt?	1	2	3	4
22. Haben Sie sich Sorgen gemacht?	1	2	3	4
23. Waren Sie reizbar?	1	2	3	4
24. Fühlten Sie sich niedergeschlagen?	1	2	3	4
25. Hatten Sie Schwierigkeiten, sich an Dinge zu erinnern?	1	2	3	4
26. Hat Ihr körperlicher Zustand oder Ihre medizinische Behandlung Ihr Familienleben beeinträchtigt?	1	2	3	4
27. Hat Ihr körperlicher Zustand oder Ihre medizinische Behandlung Ihr Zusammensein oder Ihre gemeinsamen Unternehmungen mit anderen Menschen beeinträchtigt?	1	2	3	4
28. Hat Ihr körperlicher Zustand oder Ihre medizinische Behandlung für Sie finanzielle Schwierigkeiten mit sich gebracht?	1	2	3	4

Bitte kreisen Sie bei den folgenden Fragen die Zahl zwischen 1 und 7 ein, die am besten auf Sie zutrifft

29. Wie würden Sie insgesamt Ihren Gesundheitszustand während der letzten Woche einschätzen?

1	2	3	4	5	6	7
sehr schlecht						ausgezeichnet

30. Wie würden Sie insgesamt Ihre Lebensqualität während der letzten Woche einschätzen?

1	2	3	4	5	6	7
sehr schlecht						ausgezeichnet

© QLQ-C30 Copyright 1995 EORTC Quality of Life Group. Alle Rechte vorbehalten. Version 3.0
