# Supplementary material for: Comparative effectiveness trial of transoral head and neck surgery followed by adjuvant radio(chemo)therapy versus primary radiochemotherapy for oropharyngeal cancer (TopROC)
Source: BMC Cancer. 2020 Jul 29;20:701. doi: 10.1186/s12885-020-07127-2 (PMC7389683; doi:10.1186/s12885-020-07127-2)
Supplement: Supplementary file 3 — Additional file 3. [file 12885_2020_7127_MOESM3_ESM.pdf]

Visite (z.B Baseline):

Datum:

Pat.ID:

cCc

Anderson Dysphagia Inventory-Head and Neck German

1: stimme voll zu; 2: stimme zu; 3: weiß nicht; 4: stimme nicht zu; 5: stimme auf keinen Fall zu

Meine Schluckprobleme behindern mich bei der täglichen Routine.	□1 □2 □3 □4 □5
Meine Essgewohnheiten sind mir peinlich.	□1 □2 □3 □4 □5
Die Leute finden es schwer, etwas Passendes für mich zu kochen.	□1 □2 □3 □4 □5
Das Schlucken fällt mir am Ende eines Tages schwer.	□1 □2 □3 □4 □5
Ich fühle mich nicht gehemmt wenn ich esse.	□1 □2 □3 □4 □5
Mein Schluckproblem belastet mich.	□1 □2 □3 □4 □5
Das Schlucken fällt mir sehr schwer.	□1 □2 □3 □4 □5
Wegen meines Schluckproblems gehe ich nicht mehr aus.	□1 □2 □3 □4 □5
Wegen meines Schluckbeschwerden musste ich Einkommeinseinbußen hinnehmen.	□1 □2 □3 □4 □5
Beim Essen brauch ich wegen meiner Schluckprobleme länger.	□1 □2 □3 □4 □5
Die Leute fragen mich, warum ich Dieses und Jenes nicht essen kann.	□1 □2 □3 □4 □5
Andere finden mein Essproblem als störend.	□1 □2 □3 □4 □5
Ich muss husten, wenn ich versuche zu trinken.	□1 □2 □3 □4 □5
Meine Schluckbeschwerden schränken mich im sozialen Umgang und im Privatleben ein.	□1 □2 □3 □4 □5
Ich kann jederzeit mit meinen Freunden, Nachbarn und Verwandten zum Essen ausgehen.	□1 □2 □3 □4 □5
Ich schränke die Nahrungsaufnahme wegen meiner Schluckbeschwerden ein.	□1 □2 □3 □4 □5
Wegen meiner Schluckprobleme kann ich mein Gewicht nicht halten.	□1 □2 □3 □4 □5
Ich habe wegen meiner Schluckprobleme ein niederes Selbstwertgefühl	□1 □2 □3 □4 □5
Es fühlt sich immer so an, als ob ich riesige Bissen verschlucke.	□1 □2 □3 □4 □5
Wegen meiner Essgewohnheiten fühle ich mich ausgeschlossen.	□1 □2 □3 □4 □5
