# Supplementary material for: Comparative effectiveness trial of transoral head and neck surgery followed by adjuvant radio(chemo)therapy versus primary radiochemotherapy for oropharyngeal cancer (TopROC)
Source: BMC Cancer. 2020 Jul 29;20:701. doi: 10.1186/s12885-020-07127-2 (PMC7389683; doi:10.1186/s12885-020-07127-2)
Supplement: Supplementary file 4 — Additional file 4. [file 12885_2020_7127_MOESM4_ESM.pdf]

Visite (z.B Baseline):
Datum:
Pat.ID:

Soziodemographischer Bogen

A1 geb

Geburtsdatum (Jahr) Jahr ☐☐☐☐

geschl **Geschlecht** ❑ weiblich ❑ männlich

nat1 **Nationalität** ❑ deutsch ❑ anderes: ____________________ nat2

fam1 **Familienstand**

ledig (nie verheiratet)	verheiratet (mit dem Ehepartner zusammenlebend)	verheiratet (in Trennung lebend)	geschieden	verwitwet	
❑	❑...seit ➔	❑...seit ➔	❑...seit ➔	❑...seit ➔	✎__________ (Datum) fam2

part1 **Haben Sie zur Zeit einen festen Partner?** ❑ ja... ❑ nein... seit ➔ ✎__________ (Datum) part2

kindz **Wie viele Kinder haben Sie?** __________ Anzahl ❑ keine Kinder

A2 **Wie viele Personen leben insgesamt in Ihrem Haushalt, Sie selbst eingeschlossen?**
Zählen Sie dabei bitte auch Kinder mit und tragen Sie die Anzahl ein.

wohn1 insgesamt ✎________ Personen, davon ➔ ✎________ 2 unter 18 Jahre alt

A3 **Wie hoch ist das monatliche Nettoeinkommen Ihres Haushalts insgesamt?**
Nettoeinkommen: Die Summe aus Lohn/Gehalt/Einkommen usw., nach Abzug von Steuern und Sozialabgaben.

netto
- ❑ bis unter 500 €
- ❑ 500 € bis unter 1000 €
- ❑ 1000 € bis unter 2000 €
- ❑ 2000 € bis unter 3000 €
- ❑ 3000 € bis unter 4000 €
- ❑ 4000 € und mehr

A4 haupt **Sind Sie Hauptverdiener / Hauptverdienerin Ihres Haushaltes?** ❑ ja ❑ nein

A5 schul1 **Welches ist Ihr <u>höchster</u> Schulabschluss?**

- ❑ ohne Schulabschluss abgegangen
- ❑ Haupt-/Volksschulabschluss
- ❑ Realschulabschluss/Mittlere Reife
- ❑ Abschluss der Polytechnischen Oberschule 10. Klasse (vor 1965: 8. Klasse)
- ❑ Fachhochschulreife
- ❑ Allgemeine/fachgebundene Hochschulreife/Abitur
- ❑ andere: ✎____________________ schul2

A6 ausb1 **Welches ist Ihr <u>höchster</u> berufsqualifizierender Abschluss?**

- ❑ kein berufsqualifizierender Abschluss
- ❑ abgeschlossene Lehre (beruflich-betriebliche Ausbildung)
- ❑ Handelsschule/Berufsfachschule (beruflich-schulische Ausbildung)
- ❑ Abschluss an Fachschule, Meister-, Technikerschule, Berufs- oder Fachakademie
- ❑ Fachhochschulabschluss, Ingenieurschule
- ❑ Hochschulabschluss
- ❑ andere: ✎____________________ ausb2

A6 **Sind Sie zur Zeit erwerbstätig?**
(Bitte auch ausfüllen, wenn Sie zur Zeit krankgeschrieben sind.)

w1_ty
- ❑ ja, Vollzeit
- ❑ ja, Teilzeit, und zwar ______ Stunden/Woche w2_ty
- ❑ nein, arbeitslos/erwerbslos seit ____.____.________ w3_ty
- ❑ nein, in Ausbildung/Umschulung
- ❑ nein, in Rente wegen verminderter Erwerbsfähigkeit seit ____.____.________ w4_ty
- ❑ nein, in Altersrente
- ❑ sonstiges: ____________________________________ w5_ty

A7 **Welche berufliche Position nahmen Sie vor Ihrer Erkrankung ein?**
(Falls Sie arbeitslos, berentet oder aus anderen Gründen erwerbsunfähig waren bzw. sind, geben Sie bitte Ihre **letzte Position** an.)

arb **Arbeiter(in):**	ang **Angestellte(r)** mit	bea **Beamtin/Beamter**
❑ ungelernte(r)	❑ einfacher Tätigkeit	❑ im einfachen Dienst
❑ angelernte(r)	❑ schwieriger Tätigkeit	❑ im mittleren Dienst
❑ Facharbeiter(in)	❑ leitender Tätigkeit	❑ im gehobenen Dienst
❑ Vorarbeiter(in)		❑ im höheren Dienst
❑ Meister(in)		

sel **Selbständige(r):**
- ❑ selbständige(r) Landwirt(in)
- ❑ Akademiker(in) im freien Beruf
- ❑ Selbständige(r) im Handel, Gewerbe, Handwerk, Industrie, Dienstleistung
- ❑ Mithelfende(r) im Familienbetrieb

➔ Anzahl Mitarbeiter(innen): ✎ ________ selz

son **Sonstiger, hier nicht aufgeführter Beruf:** ✎ ____
